# Treatment of infertility in women with polycystic ovary syndrome: approach to clinical practice

**DOI:** 10.6061/clinics/2015(11)09

**Published:** 2015-11

**Authors:** Anderson Sanches Melo, Rui Alberto Ferriani, Paula Andrea Navarro

**Affiliations:** Universidade de São Paulo, Faculdade de Medicina de Ribeirão Preto, Departamento de Ginecologia e Obstetrícia, Ribeirão Preto/SP, Brazil

**Keywords:** Polycystic Ovary Syndrome, Infertility, Clomiphene Citrate, Ovarian Drilling, *In Vitro* Fertilization

## Abstract

Polycystic ovary syndrome represents 80% of anovulatory infertility cases. Treatment initially includes preconception guidelines, such as lifestyle changes (weight loss), folic acid therapy to prevent the risk of fetal neural tube defects and halting the consumption of tobacco and alcohol. The first-line pharmacological treatment for inducing ovulation consists of a clomiphene citrate treatment for timed intercourse. The second-line pharmacological treatment includes the administration of exogenous gonadotropins or laparoscopic ovarian surgery (ovarian drilling). Ovulation induction using clomiphene citrate or gonadotropins is effective with cumulative live birth rates of approximately 70%. Ovarian drilling should be performed when laparoscopy is indicated; this procedure is typically effective in approximately 50% of cases. Finally, a high-complexity reproduction treatment (*in vitro* fertilization or intracytoplasmic sperm injection) is the third-line treatment and is recommended when the previous interventions fail. This option is also the first choice in cases of bilateral tubal occlusion or semen alterations that impair the occurrence of natural pregnancy. Evidence for the routine use of metformin in infertility treatment of anovulatory women with polycystic ovary syndrome is not available. Aromatase inhibitors are promising and longer term studies are necessary to prove their safety.

## INTRODUCTION

Polycystic ovary syndrome (PCOS) is an endocrine and reproductive disorder with a prevalence ranging from 5% 1 to 13% 2 in women of reproductive age. PCOS is the primary cause of hyperandrogenism and oligo-anovulation at the reproductive age and is often associated with infertility 3 and clinical and metabolic disorders 4.

The prevalence of infertility in women with PCOS varies between 70 and 80%. According to the American Society for Reproductive Medicine, the evaluation of infertility in women with PCOS or other causes of subfertility should start after six months of attempting pregnancy without success if the couple has regular sexual intercourse (2 to 3 times/week) without using contraceptive methods 7. To optimize the efficacy of the treatment of infertile women with PCOS, evaluations of tubal patency (hysterosalpingography or laparoscopy with chromotubation) and semen analysis (spermogram) are mandatory before deciding on treatment. However, tubal patency evaluation may not be necessary prior to initiating clomiphene citrate (CC) treatment. Notably, if a patient is resistant to this drug and/or requires the use of gonadotropins and/or presents with other causes of infertility, a tubal patency evaluation becomes mandatory prior to initiating the therapeutic treatment of infertility 8.

The principle infertility treatment initially includes preconception guidelines and the use of drugs to induce mono- or bifollicular ovulation. Other therapeutic modalities may also be employed, such as exogenous gonadotropins or laparoscopic ovarian drilling, which are considered to be second-line treatments, or *in vitro* fertilization (IVF), which is a third-line treatment 9. Thus, the choice of the most appropriate treatment depends on the patient's age, presence of other factors associated with infertility, experience and duration of previous treatments and the level of anxiety of the couple.

### Non-pharmacological measures

#### Change in lifestyle and counseling of pregnancy complications in women with PCOS

Lifestyle change is considered the first-line treatment for infertility in obese women with PCOS. Preconception counseling, administering folic acid to reduce the risk of fetal neural tube defects, encouragement of physical activity and identification of risk factors, such as obesity, tobacco use and alcohol consumption, should be performed. A 5 to 10% loss in body weight over a period of six months regardless of body mass index may be associated with improvement in central obesity, hyperandrogenism and ovulation rate 9. However, no studies with the proper methodology have assessed the live birth rate, which is the primary reproductive outcome 10.

Obese women with PCOS may have an increased risk of congenital anomalies (heart and neural tube defects), gestational diabetes mellitus [odds ratio (OR) 2.94; 95% confidence interval (CI): 1.70-5.08], hypertensive disorders during pregnancy (OR 3.67; 95% CI: 1.98-6.81) [mainly preeclampsia (OR 3.47; 95% CI: 1.95-6.17)], miscarriages, preterm births (OR 1.75; 95% CI: 1.16-2.62), the need for intensive unit care (OR 2.31; 95% CI: 1.25-4.26), increased perinatal mortality (OR 3.07; 95% CI: 1.03-9.21) 11,12 and Caesarean delivery (OR 1.74; 95% CI: 1.38-2.11) 12. The risk for preterm births and preeclampsia appears to be associated with maternal hyperandrogenism 13.

In addition to improving reproductive and metabolic factors, the reduction in body weight may be associated with reduced incidence of complications during pregnancy and the neonatal period. In this context, lifestyle change should be the first choice for weight loss because medications to reduce weight could have side effects and bariatric surgery may be associated with preterm and small for gestational age births 14.

### Pharmacological measures

#### First-line treatment: Clomiphene citrate

In anovulatory women with PCOS defined according to the Rotterdam consensus (includes all phenotypes except the one defined by the association of hyperandrogenism with ultrasound (US) findings), CC treatment is the first choice for ovulation induction 9,15. This drug is an estrogen receptor modulator (it can act as an estrogen agonist or antagonist) and its mechanism of action is controversial but can be explained as follows. In physiological menstrual cycles, low levels of estrogen promote negative feedback in the hypothalamus and pituitary gland and inhibit the endogenous secretion of gonadotropin during the early follicular phase. When CC is administered in this phase of the cycle, it competes with estrogen for its receptors in the hypothalamus and pituitary, which will block the negative feedback mechanism. Consequently, increased levels of endogenous gonadotropins are released and the dominant follicle is recruited (follicle that has the highest number of follicle-stimulating hormone (FSH) receptors) between the sixth and ninth day of the menstrual cycle 16.

The advantages of CC use are low cost, oral administration, few side effects (flushing, headache, visual disturbances and abdominal discomfort), the induction of monofollicular development in most cases 16 and a low rate of multiple gestations (2 to 13%) 17. The initial dose is 50 mg/day for five days (starting between the second and fifth day of the menstrual cycle) and may be increased to 150 mg/day 17,18; however, doses greater than 100 mg/day usually do not offer additional benefits (may be useful in obese women) 18. The ovulation rate may reach 75 to 80% 19 with a conception rate of 22% per cycle 20 and a cumulative pregnancy rate between 60 and 70% in six cycles 9. There is no evidence that the administration of human chorionic gonadotropin (hCG) in the mid-cycle increases ovulation rates (OR 0.99; 95% CI: 0.36-2.77) or clinical pregnancy (OR 1.02; 95% CI: 0.56-1.89) 21,22. CC treatment should be limited to six ovulatory cycles and US monitoring is not mandatory (it is recommended only in the first ovulatory cycle to adjust the dose based on the ovarian follicular growth and development and for endometrial assessment) 17,18. Additional cycles of ovulation induction with CC (maximum of twelve cycles) may be individually evaluated based on the cost-effectiveness and age of women and after discussion with the couple 9. The incidence of ovarian hyperstimulation syndrome (OHSS; increased capillary permeability with consequent third-space fluid sequestration and hemoconcentration) associated with the use of CC is low, approximately 1 to 6% 17,23.

Approximately 15% of women with PCOS do not respond to the maximum dose of CC and are considered resistant to this medication. Due to the anti-estrogenic effect of this drug, endometrial proliferation may be inappropriate, which decreases the chance of embryo implantation. Moreover, this effect can also change the cervical mucus characteristics with a consequent reduction in sperm penetration 17,23. If the patient does not ovulate after the use of CC, gonadotropins for timed intercourse or ovarian drilling are the next steps to manage anovulatory infertile women with PCOS 9.

In practice, CC treatment can initiate the menstrual cycle as early as the second day. Classically, this drug treatment has been initiated between the third and fifth day of the menstrual cycle and maintained for 5 days. Ovulation typically occurs seven days after the last CC tablet is taken. Seven days after the probable date of ovulation, follicular rupture can be confirmed by progesterone levels greater than 3 ng/dL (evaluated only at the beginning of the treatment to verify the response to CC when US is unavailable) and pregnancy can be confirmed by measuring the blood beta fraction of human chorionic gonadotropin (βhCG) 7 days after the progesterone measurement. The couple should maintain their usual frequency of sexual intercourse, including during the fertile period. This protocol is ideal for primary healthcare centers with limited subsidiary resources.

Where US is available, CC treatment should be initiated between the third and fifth day of the menstrual cycle and the couple should abstain from intercourse (this is not a mandatory measure) until the tenth day of the cycle (when the presence of dominant follicles with a mean diameter of 10 mm or more is assessed via US). Sexual activity is allowed if the patient presents with monofollicular or bifollicular development. The goal of sexual abstinence until the tenth day of the cycle is to minimize the risk for multiple gestations.

Couples with infrequent sexual intercourse may experience some benefit from the use of kits for ovulation monitoring (urinary luteinizing hormone excretion); however, this technique can underestimate the fertile window. The evaluation of cervical mucus throughout the menstrual cycle demonstrated similar efficacy to urinary kits for monitoring the ovulation and high rates of false positives in cycles are noted using the hCG 24. Thus, this method has not been routinely used in clinical practice, mainly when US is available.

#### Second-line treatment: Gonadotropins

The second-line pharmacological treatment of infertility in anovulatory women with PCOS includes the use of gonadotropins [recombinant follicle-stimulating hormone (FSHr) or human menopausal gonadotropin (HMG)] for timed intercourse or intrauterine insemination (IUI) 9. Due to the higher cost of this therapeutic modality, an evaluation of the tubal patency is recommended prior to initiating the ovarian stimulation with gonadotropins if this procedure was not performed prior to initiating CC treatment. If the fallopian tube is opened and the sperm concentration is suitable for *in vivo* fertilization, the ovarian stimulation begins with low doses of gonadotropins (37.5 to 75 IU/day or every other day) to achieve monofollicular growth and reduce the risk of complications (OHSS and multiple gestation) 25. US monitoring of the follicular growth (follicular diameter measurement) is mandatory in this case and the endogenous secretion of gonadotropins does not need to be inhibited with gonadotropin-releasing hormone analogues (GnRH-a) during the timed intercourse cycles. The administration of hCG (used to simulate the endogenous peak of luteinizing hormone for final oocyte maturation and ovulation triggering) is unnecessary because it does not increase the probability of conception during ovulation induction cycles for timed intercourse 21. It is important to note that if gonadotropin is chosen as the treatment option, the IUI has a higher likelihood of successful pregnancy compared with timed intercourse in patients with subfertility 26.

The IUI is performed with the same dose of gonadotropins recommended for timed intercourse (combined or not with clomiphene). However, for this treatment modality, the recombinant hCG is administered for final oocyte maturation when the dominant follicle has a mean diameter of 17 to 18 mm via US examination and capacitated sperm can be injected into the uterine cavity 36 hours later. Beta hCG is measured 14 days later to confirm pregnancy 25.

Sperm capacitation must be evaluated to perform the low-complexity treatment (semen evaluation after preparation to estimate the number of sperm with progressive motility, which includes those that theoretically have the ability to ascend the female reproductive tract *in vivo* and fertilize the egg in the fallopian tube). Thus, the semen is centrifuged and the concentration of capacitated sperm recovered is measured as follows: >10 million recovered motile sperms (any infertility treatment is viable); >5 million (IUI, *in vitro* fertilization (IVF) or intra-cytoplasmic sperm injection (ICSI) may be performed); between 1 and 5 million (IVF or ICSI may be performed); and <1 million (only ICSI can be performed) 27,28. It is worth noting that if the patient presents with bilateral tubal occlusion in the initial assessment, sperm capacitation is only performed to evaluate the possibility of performing IVF or ICSI 8.

The use of gonadotropins for timed intercourse is associated with an ovulation rate of approximately 70%, a clinical pregnancy rate of 20% per cycle and a multiple live birth rate of 5.7% 9. Due to the cost of the treatment, the need for regular monitoring of the follicular development via ultrasound and the higher pregnancy rates with IUI, the use of gonadotropin is not routine for timed intercourse. Instead, this medication is used in IUI 26 or high-complexity treatments (IVF or ICSI) 9.

#### Second-line treatment: Laparoscopic ovarian surgery

This therapeutic modality is also considered a second-line treatment for the infertility of women with PCOS. However, because it is an invasive method that requires general anesthesia and has a higher cost and potential complications, this technique should be used in cases of anovulatory women with CC-resistant PCOS who require laparoscopy for another reason (pelvic pain, adnexal mass, etc.). This technique can be performed using monopolar electrocautery or laser techniques, with both exhibiting a similar efficacy and the goal is between 4 and 10 punctures because a larger number may favor the development of premature ovarian failure 25,29. The mechanism of action of ovarian drilling in the treatment of infertility in women with PCOS is suggested to be based on the decreased secretion of androgens and consequent reduction of peripheral aromatization of these compounds into estrone. Furthermore, the follicular microenvironment becomes more estrogenic, which facilitates follicular growth 30. Regarding the efficacy of ovarian drilling, observational studies demonstrated that the ovulation rate was between 54 and 76% in the 6 months after the procedure and 33 and 88% in the 12 months after the procedure. During these periods, the spontaneous pregnancy rate ranged between 28 and 56% and 54 and 70%, respectively 31.

If the patient does not present with ovulatory cycles at three months after ovarian drilling, then the procedure should be combined with CC treatment. The use of gonadotropins should be considered after 6 months of anovulatory cycles following the ovarian drilling procedure. Ovarian drilling should not be indicated as a treatment for menstrual irregularity, metabolic complications or hyperandrogenism in PCOS 29.

Ovarian drilling has some advantages compared with gonadotropin treatment because it is associated with a lower multiple gestation rate (OR 0.13; 95% CI: 0.03 to 0.52; *p*=0.004; I(2)=0%; 5 trials; n=166) 29 and does not require US monitoring of follicular development 9. However, the long-term impact of ovarian drilling on the ovarian reserve/ovarian function remains unknown 29.

Due to the high cost of the procedure, the need for hospitalization, general anesthesia and higher complications risks, ovarian drilling presents low cost effectiveness compared with gonadotropin plus timed intercourse. Moreover, the lack of standardization of the surgical technique and the absence of studies that have evaluated the repercussions of long-term of ovarian drilling demonstrate that this procedure should not be routinely performed but should only be considered as second line of therapy in women with PCOS who will be undergoing laparoscopy for another reason (adnexal mass or pelvic pain, for example). Additionally, ovarian drilling could be an alternative before the assisted reproduction treatment (ART) in individuals without financial conditions for the realization of ART and those who are resistant to CC.

#### Third-line treatment: *in vitro* fertilization

*In vitro* fertilization represents the third-line treatment for infertility in women with PCOS 9. However, if the initial assessment demonstrates a bilateral tubal occlusion and/or concentration of recovered motile sperm less than or equal to 5 million, this treatment becomes the first option along with lifestyle changes. The risk of OHSS is the main complication of the highly complexity treatment in women with PCOS. Thus, to minimize this side effect, ovarian stimulation should be initiated with low doses of gonadotropins (100 to 150 IU of FSHr) and the pituitary should be suppressed with a gonadotropin-releasing hormone (GnRH) antagonist because this method is associated with a reduced risk of OHSS compared with an agonist (29 randomized control trials (RCTs); OR 0.43; 95% CI: 0.33 to 0.57) 32. If the patient presents with clinical and ultrasound signs of OHSS, final oocyte maturation should be performed with a GnRH agonist and embryos should be frozen and transferred in a subsequent cycle 33,34. Infertile women with PCOS may present with better general oocyte and embryo quality rates; however, the clinical pregnancy and live birth rates are similar to those observed in normo-ovulatory women without PCOS 35.

### Metformin

Although metformin is associated with better clinical pregnancy rates (positive beta hCG) (pooled OR 2.31; 95% CI: 1.52 to 3.51; 8 trials; 707 women), there is no evidence of better live birth rates (the main variable used to evaluate the effectiveness of a treatment for infertility) when this drug is used alone (pooled OR 1.80, 95% CI: 0.52 to 6.16; 3 trials; 115 women) or in combination with CC (pooled OR 1.16; 95% CI: 0.85 to 1.56; 7 trials; 907 women) 36. From a reproduction standpoint, there is also no benefit for its use in short (less than four weeks) or long (more than four weeks) periods prior to starting CC treatment in women with PCOS. Therefore, the use of metformin should be restricted to the treatment of glucose intolerance or type 2 diabetes in women with PCOS and should not be used to induce ovulation 9,36.

However, in women with PCOS receiving low doses of gonadotropins for timed intercourse, metformin administration can double the clinical pregnancy rate (OR 2.25; 95% CI: 1.50 to 3.38; *p*<0.001; 7 trials) and the live birth rate (OR 1.94; 95% CI: 1.10 to 3.44; *p*=0.020; 2 trials). Moreover, this practice can reduce the cancellation rate due to ovarian hyperresponsiveness by approximately 60% (OR 0.41; 95% CI: 0.24 to 0.72; *p*=0.002; 7 trials), the number of days of stimulation (mean difference (MD)=-3.28; 95% CI: -6.23 to 0.32; *p*=0.030; 6 trials) and the dose of gonadotropins (MD=-306.62; 95% CI: -500.02 to -113.22; *p*=0.002; 7 trials) in low-complexity cycles. However, the use of metformin is not related to a reduction in the multiple pregnancy rate (OR 0.32; 95% CI: 0.08 to 1.23; *p*=0.100; 3 trials), a change in the miscarriage rate (OR 0.47; 95% CI: 0.14 to 1.54; *p*=0.210; 5 trials) or OHSS (OR 0.56; 95% CI: 0.26 to 1.21; *p*=0.140; 5 trials). Notably, no conclusive data are available on the appropriate dose and time (pre-treatment or during gonadotropin treatment) for the use of metformin during timed intercourse with gonadotropins 37.

For assisted reproduction cycles, metformin use prior to or during ovarian stimulation with gonadotropins in IVF/ICSI cycles is also not associated with better clinical pregnancy or live birth rates; however, metformin may reduce the risk of OHSS 38,39 and miscarriage and improve the implantation rate because metformin may act directly on the endometrium 39 and promote better reproductive outcomes (data not confirmed) in women with PCOS 40. However, as previously mentioned, the use of a GnRH antagonist combined with ovarian stimulation with gonadotropins in women with PCOS and the induction of final ovarian maturation with a GnRH agonist with subsequent embryo cryopreservation are more effective strategies to prevent OHSS regardless of metformin use 33. Thus, the routine use of metformin in cycles of ovarian stimulation for IVF in women with PCOS is not recommended except in the presence of a disorder in glucose metabolism 9.

#### Aromatase inhibitors

Although aromatase inhibitors have been used in women with PCOS as an alternative method to avoid the anti-estrogenic effect of CC on the endometrium, these compounds are not typically used in clinical practice to treat infertility in these patients. Their mechanism of action is based on reducing the peripheral conversion of androgens to estrogens in ovarian granulosa cells by blocking aromatase. Consequently, a decrease in estrogen serum levels and in its negative feedback in the hypothalamus and pituitary gland is noted, resulting in increased endogenous gonadotropin release 41.

The effectiveness of aromatase inhibitors in the treatment of PCOS remains controversial. A meta-analysis investigated 78 studies on the use of these medications in the infertility treatment of women with PCOS. Of these studies, 13 RCTs met the inclusion criteria. Six studies compared the use of letrozole versus CC and found that letrozole presented with a higher ovulation rate/patient (OR 2.90; 95% CI: 1.72- 4.88; *p*<0.0001); however, no significant differences in the rate of ovulation per cycle or better pregnancy, live birth, multiple pregnancy or miscarriages rates were noted. Letrozole also did not obtain better results regarding clinical pregnancy or live birth rates compared with placebo or CC + metformin in women with CC-resistant PCOS. The results of the comparison of the effects of letrozole and anastrozole on ovulation and pregnancy rates in women with CC-resistant PCOS are controversial 41.

In contrast, another recent meta-analysis reviewed 26 studies that evaluated the use of letrozole in women with PCOS. The use of letrozole in cycles for timed intercourse was associated with higher live birth (nine studies; OR 1.63; 95% CI: 1.31 to 2.03; n=1783; I^2^=3%) and clinical pregnancy rates (fourteen studies; OR 1.32; 95% CI: 1.09 to 1.60; n=2066; I^2^=25%) compared with CC treatment; however, this evidence was poor. Studies comparing the use of letrozole *versus* ovarian drilling revealed no differences in live birth, clinical pregnancy or OHSS rates. The administration of letrozole for 5 or 10 days at a dose of 5 or 7.5 mg/day displayed similar clinical pregnancy rates 42. A recent study found that the use of letrozole was associated with higher live birth rates and ovulation among 750 infertile women with polycystic ovary syndrome compared with clomiphene 43.

From a practical standpoint, the use of aromatase inhibitors may be an option before IVF/ICSI after counseling and the consent of the couple in specific cases of women with CC-resistant PCOS without other infertility factors and for whom the high-complexity treatment is cost-prohibitive 41. The recommended dose of letrozole is 5 to 10 mg/day for 5 to 10 days.

The principle infertility treatment includes lifestyle changes. The first-line drug treatment to induce ovulation consists of CC with timed intercourse. The second-line treatment consists of the exogenous administration of gonadotropins or laparoscopic ovarian surgery in cases where laparoscopy is indicated. The third-line treatment consists of IVF/ICSI, which is indicated when the previous interventions fail; this treatment can also be the first choice in cases of bilateral tubal occlusion or semen alterations that impair the occurrence of natural pregnancy. There is no evidence for the routine use of metformin in infertility treatment of anovulatory women with PCOS. Aromatase inhibitors are promising, and long-term studies are necessary to prove their safety.
